# Long-Read Sequencing Identified a *PKD1* Gene Conversion in ADPKD Rather Than the False-Positive Exon Deletion Indicated by WES and MLPA

**DOI:** 10.1155/2024/7225526

**Published:** 2024-09-20

**Authors:** Xueping Qiu, Xin Jin, Jin Li, Yuanzhen Zhang, Jianhong Ma, Fang Zheng

**Affiliations:** ^1^ Center for Gene Diagnosis & Department of Laboratory Medicine Zhongnan Hospital of Wuhan University, Wuhan 430071, China; ^2^ Hubei Clinical Research Center for Prenatal Diagnosis and Birth Health, Wuhan 430071, China

**Keywords:** ADPKD, gene conversion, long-read sequencing, *PKD1*, WES

## Abstract

Whole exome sequencing (WES) has become an increasingly common technique for identifying the genetic cause of Mendelian genetic diseases. However, it may fail to detect the complex regions of the genome. Here, we investigated the genetic etiology of a pedigree with autosomal dominant polycystic kidney disease (ADPKD) using a combination of WES, multiplex ligation-dependent probe amplification (MLPA), Sanger sequencing, and long-read sequencing (LRS). Initially, WES of the proband revealed a heterozygous variant c.7391G>C in *PKD1* Exon 18, along with a heterozygous deletion of the 17th and 18th exons of *PKD1* detected by exome-based copy number variation (CNV) analysis. MLPA confirmed the *PKD1* heterozygous deletion of Exon 18. Except for c.7391G>C, Sanger sequencing identified four other heterozygous variants (c.7278T>C, c.7288C>T, c.7344C>G, and c.7365C>T) in Exon 18 of *PKD1*. Subsequently, LRS uncovered seven clustered substitution variants (c.7209+28C>T, c.7210-16C>T, c.7278T>C, c.7288C>T, c.7344C>G, c.7365C>T, and c.7391G>C), with six of them omitted by WES due to interference from *PKD1* pseudogenes. Combining LRS results with cosegregation of the pedigree analysis, we found these variants were in *cis* and converted from *PKD1* pseudogenes, covering a region of at least 282 bp. Notably, the paralogous sequence variants of c.7288C>T introduced a premature stop codon of *PKD1*, leading to a function loss, and were classified as pathogenic (PVS1+PS4+PM2) according to the ACMG/AMP guideline. Our study highlights the limitations of WES/MLPA and the importance of utilizing complementary tools like LRS for comprehensive variant detection in *PKD1*.

## 1. Introduction

Polycystic kidney disease (PKD) is genetically heterogeneous with patterns of autosomal dominant inheritance and autosomal recessive inheritance [1]. Autosomal dominant polycystic kidney disease (ADPKD) is the most common inherited adult kidney disease, which is characterized by gradually growing renal cysts, enlargement of kidney volume, and eventually end-stage kidney disease (ESKD) [2]. ADPKD is primarily caused by the mutation of *PKD1* and *PKD2*, which account for approximately 85% and 15%, respectively. Moreover, several novel causative genes have recently been identified in atypical ADPKD, such as *DNAJB11* and *GANAB* [2]. Additionally, the increasing genes associated with an overlapped ADPKD-like phenotype have been recognized, such as *HNF1B* and *OFD1* [1].

The genetic analysis of ADPKD seems to be more challenging than other monogenetic diseases. Firstly, *PKD1* and *PKD2* are highly variable genes, and *PKD1* contains 46 exons with a coding length of 12912 bp (NM_001009944.3), spanning a genomic region of about 50 kb. Moreover, *PKD1* had six pseudogenes with sequence identities of approximately 98% [2]. Therefore, the large size, high homology, high complexity, and high GC content of *PKD1* result in difficulty in detecting mutations within this gene [3]. Additionally, several causal genes without mutation hotspots and the overlapped phenotype made the genetic diagnosis of ADPKD more complex.

To avoid inadvertently sequencing the *PKD1* pseudogene, long-range PCR (LR-PCR) amplification is used to generate locus-specific templates. Rossetti et al. [4] used five LR-PCR followed by nested PCR to screen the whole region of *PKD1* and yielded a high diagnostic rate of 80%–90% in selected patients, but it is costly and labor-intensive. Although single nucleotide variants (SNVs) and short insertion and deletion (indels) are the predominant types of variants in *PKD1* and *PKD2*, large deletion and duplication have also been identified. Multiplex ligation-dependent probe amplification (MLPA) is a valuable technology for detecting the deletions or duplications in the *PKD1* and *PKD2* genes, especially in cases where PCR-sequencing yields negative results [5]. Nowadays, whole exome sequencing (WES) has emerged as a powerful tool for investigating multiple genes simultaneously, offering advantages in terms of cost-effectiveness, timesaving, and laborsaving [6, 7]. However, as one of the members of short-read sequencing (SRS) technology, its inherent drawbacks cause the decreasing sensitivity and specificity for ADPKD. Firstly, the short read of SRS may pose challenges in accurately mapping to the genomic reference sequence, complicating the differentiation between genuine gene and pseudogene sequences in homologous regions [8]. Additionally, SRS may fail to detect the regions with high GC content and complex structure because of amplification difficulties in these fragments [9].

The rapidly developing third-generation single molecular long-read sequencing (LRS) may overcome some shortcomings of SRS as it advances in detecting genomic rearrangements, especially in repetitive or complex genomic regions [10]. Moreover, the LRS enables targeted library preparation without requiring PCR amplification. Within the long sequencing reads (typically 1000 bp or longer), researchers can conveniently distinguish the sequence of genuine genes from their pseudogenes, discover the accurate breakpoint regions, and so on [8, 11].

In this study, we reported a causative gene conversion of *PKD1* in an ADPKD family using LRS, which has been misjudged as exon deletion by WES and MLPA. To our knowledge, this is the first report to identify the causative gene conversion in relative regions of the *PKD1* gene by LRS.

## 2. Materials and Methods

### 2.1. Patient Samples

**Figure 1:**
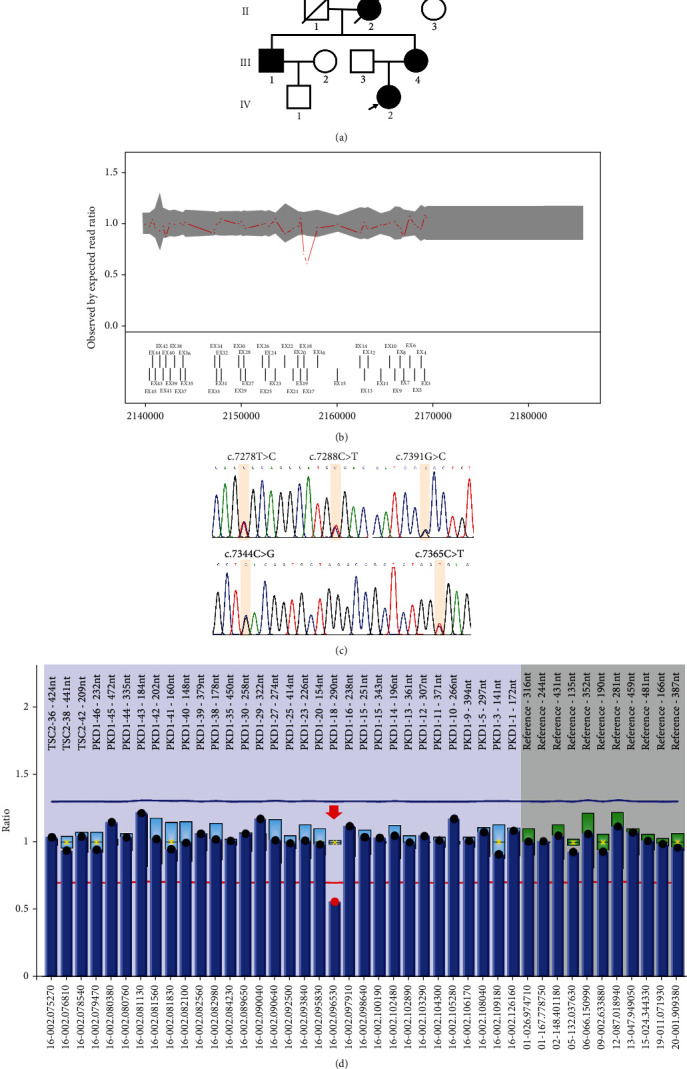
Pedigree and variant analysis of the proband. (a) Pedigree chart of the family. Proband is indicated by an arrow (IV-2). Two suspected patients (III-1 and III-4) and two unaffected individuals (II-3 and III-3) participated in follow-up studies. (b) Exome-based CNV analysis identified the heterozygous deletion of Exons 17 and 18 in *PKD1* using the data of WES. (c) The heterozygous of variants in Exon 18 of *PKD1* were identified by Sanger sequencing. (d) MLPA-P351 identified a heterozygous deletion in Exon 18 of *PKD1*, as indicated by the red arrow.

In this study, five individuals from an ADPKD family were enrolled (Figure 1(a), IV-2, III-1, III-3, III-4, and II-3). The family history was taken from the proband, and a pedigree was prepared to document each patient with a suspected phenotype of ADPKD. The proband found multiple renal cysts in both kidneys by ultrasonography and was clinically diagnosed as PKD at the age of 24 years old. And two affected family members (III-1 and III-4) were required renal dialysis treatment before the age of 50 due to renal cysts.

The genomic DNA of three patients and two unaffected family members was extracted from peripheral blood leukocytes using standard methods (BGI, Wuhan, China). The total RNA of the proband was obtained from blood leukocytes using TRIzol (Invitrogen, CA), and cDNA was generated with the cDNA Synthesis Kit (Code No. R412-01, Vazyme, Nanjing, China). Informed consents were approved by all participants. This study was approved by the Medical Ethical Committee of Zhongnan Hospital of Wuhan University and complied with the principles of the Declaration of Helsinki.

### 2.2. WES

Sheared genomic DNA of the proband was used for library preparation by the MGIEasy DNA Library Prep Kit following the manufacturer's instructions (BGI, China). Exome DNA was captured using KAPA HyperExome hybridization enrichment reagent (Roche, Switzerland) and then sequenced on MGISEQ-2000 to produce paired-end 100 bp reads. After the sequencing was completed, the reads of low quality and those contaminated with joints were removed from the original data. The clean reads were mapped to the Human Genome Reference (hg19/GRCh37). After comparison, the output file was used for sequencing coverage and in-depth analysis of the target region, SNV, and indel calls. Exonic and splice site variants with a minor allele frequency of less than 0.01 in public databases (dbSNP database [https://www.ncbi.nlm.nih.gov/snp/], gnomAD [v2.1.1, http://gnomad.broadinstitute.org], Exome Aggregation Consortium [ExAC] [http://exac.broadinstitute.org/], and 1000 Genomes [http://www.1000genomes.org/]) were selected. The exome-based copy number variation (CNV) was analyzed by the exomeDepth software [12] (https://github.com/vplagnol/ExomeDepth) to detect the variants of deletion and duplication. The pathogenicity of the variants was assessed according to the standards and guidelines of the American College of Medical Genetics and Genomics/Association of Molecular Pathology (ACMG/AMP) [13].

### 2.3. LRS

A target sequencing library was prepared using SMRTbell Express Template Preparation Kit 2.0 according to the manufacturer's instructions. Briefly, 3 *μ*g of genomic DNA was sheared to 1∼6 kb fragment by a g-Tube (#006404, Covaris) and centrifuged (6000 × g, 2 min, twice). End repair, A-tailing at the 3′ ends, and adapter ligation were performed through precapture amplification. The library was then purified and characterized for fragment size. The targeted sequence was captured by hybridization with custom capture probes ordered from BOKE Bioscience specifically for genes associated with the renal cyst phenotype, such as *PKD1*, *PKD2*, *GANAB*, *DNAJB11*, *HNF1B*, and *PKHD1*. Purified DNA fragments underwent amplification by PCR, purification, and quantification, and then they were sequenced on the Pacific Biosciences Sequel IIe following the standard protocol. Wuhan GrandOmics BioSciences Co. completed the bioinformatics analysis of SMRT sequencing.

### 2.4. Sanger Sequencing

LR-PCR was used to amplify the region of Exons 15–23 of *PKD1* from genomic DNA or cDNA samples using PrimeSTAR® HS DNA Polymerase with GC Buffer (Code No. R044Q, Takara) according to the manufacturer's protocol. For genomic DNA samples, the PCR products were purified by agarose gel extraction and sequenced on the ABI 3500Dx Genetic Analyzer (Applied Biosystems, United States) using the BigDye Terminator v3.1 Cycle Sequencing Kit (Applied Biosystems, United States) following the manufacturer's illustrations. For cDNA samples, the PCR products were purified and cloned to the pMD18-T vector (Takara) according to the manufacturer's instruction. Then, the clones were randomly chosen for plasmid extraction and sequencing analysis. The primers used in this study were obtained from the literature [14, 15] and are listed in Table 1.

### 2.5. MLPA

To validate the deletion of *PKD1* suggested by WES, a commercially available MLPA kit (P351-D1, MRC-Holland, Amsterdam, The Netherlands) was used according to the manufacturer's protocols.

## 3. Results

### 3.1. WES Identified a Heterozygous Variant and an Exon Deletion on Exon 18 of PKD1 in Proband

The WES revealed two suspected causative variants in the *PKD1* gene. The first variant identified was a heterozygous SNV, *PKD1* (NM_001009944.3): c.7391G>C (p.Arg2464Pro). This particular variant had not been described previously and was absent both in the 1000 Genomes Project and the ExAC databases. Only the gnomAD database reported a population frequency of 0.000018 for this variant. Subsequently, this variant was classified as a variant of uncertain significance (VUS: PM2) according to the ACMG/AMP criteria. Interestingly, Yu et al. [16] had also reported another missense variant (c.7391G>T [p.Arg2464Leu]) at the same code, which was also categorized as a VUS. Additionally, exome-based CNV analysis identified a heterozygous deletion spanning Exons 17 and 18 in the *PKD1* gene (Figure 1(b)), which had been classified as pathogenic (P: PVS1+PM2+PP4).

Since the c.7391G>T heterozygous variant was located on Exon 18 of *PKD1*, a heterozygous deletion of Exon 18 was also detected. MLPA and Sanger sequencing for LR-PCR products were used for further verification. In addition to c.7391G>C, Sanger sequencing identified four other heterozygous variants (c.7278T>C, c.7288C>T, c.7344C>G, and c.7365C>T) in Exon 18 of *PKD1* (Figure 1(c)). The MLPA analysis using the P351 probe indicated a heterozygous loss of Exon 18 in *PKD1* (Figure 1(d)). Unfortunately, Exon 17 of the PKD1 gene cannot be validated due to the absence of MLPA probes, as a result of its high homology with pseudogenes.

Obviously, it was conflicted that a heterozygous SNV and a heterozygous deletion were at the same region of *PKD1* since there would be homozygous variants caused by a loss of heterozygosity (LOH) due to deletion. Considering the cautionary note in the MLPA product, we regarded that single exon deletions could lead to false positive results due to the presence of SNVs; it was speculated that the deletion of Exon 18 in *PKD1* might be a false positive finding, necessitating further validation.

### 3.2. LRS Showed a Gene Conversion of PKD1 Derived From Its Pseudogene

**Figure 2:**
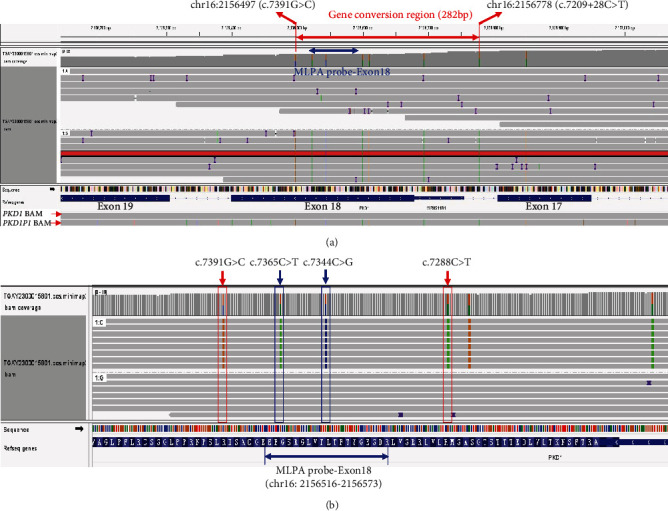
The variant analysis of *PKD1* using LRS for proband. (a) The overview variant analysis of *PKD1* in the region of Exons 17–19. The red solid rectangle indicates no deletion in Exons 17–19 of *PKD1*. The colored vertical lines of *PKD1P1* BAM displayed the distinguished variants between *PKD1* pseudogenes and genuine. The region marked by the red arrow indicated the minimum gene conversion region (282 bp) from chr16: 2156497 to chr16: 2156778. In addition, there was a heterozygous variant of c.7165T>C on Exon 17 of *PKD1* with maximum allele frequency of >0.01 based on the gnomAD v.2.1 database. Due to its high prevalence in the population, we consider c.7165T>C more of a polymorphism than a mutation. Therefore, this variant was not included in the conversion region. The region marked by a blue arrow indicated the MLPA probe region of *PKD1* gene Exon 18. (b) The main variants of the *PKD1* gene identified by LRS. The red hollow rectangle and arrows indicate the heterozygous of *PKD1*: c.7391G>C and *PKD1*: c.7288C>T. The blue hollow rectangle and arrows indicate the heterozygous of *PKD1*: c.7365C>T and *PKD1*: c.7344C>G, which was located on the MLPA probe region of *PKD1* gene Exon 18 (chr16: 2156516-2156573).

To clarify this puzzling problem, we conducted a targeted LRS on the proband's genomic DNA. The result showed no deletion of Exons 17 or 18 instead of several low-frequency heterozygous variants (gnomAD_Propmax AF <0.0001) around Exon 18 of *PKD1* (NM_001009944.3), including c.7209+28C>T (intron17), c.7210-16C>T (intron17), c.7278T>C (p.Ser2426Ser), c.7288C>T (p.Arg2430Ter), c.7344C>G (p.Leu2448Leu), c.7365C>T (p.Gly2455Gly), and c.7391G>C (p.Arg2464Pro) (Figure 2). The variant of c.7288C>T (p.Arg2430Ter) had already been reported as a pathogenic variant in the HGMD database, ClinVar database, and several literatures [17–19], which had been classified as pathogenic (P: PVS1+PS4+PM2) according to the ACMG/AMP guideline. Except for c.7391G>C (p.Arg2464Pro), the other five variants had also been evaluated as VUS. Moreover, in the advantage of the long read length for LRS, we found that these variants were present *in cis* on the same allele (Figure 2(a)). Sequence alignment indicated that all these variants (except c.7365C>T) can be perfectly matched against the *PKD1* pseudogenes *PKD1P1* (Figure 2(a)), while the flanking sequence was identical to *PKD1*. Accordingly, we speculated that a gene conversion existed between *PKD1* and pseudogenes, and the minimal converted sequence was 282 bp (Figure 2(a), chr16: 2156497-2156778, from c.7209+28 to 7391).

### 3.3. TA Clone Sequencing and Pedigree Analysis Further Validated the Gene Conversion Event of PKD1

**Figure 3:**
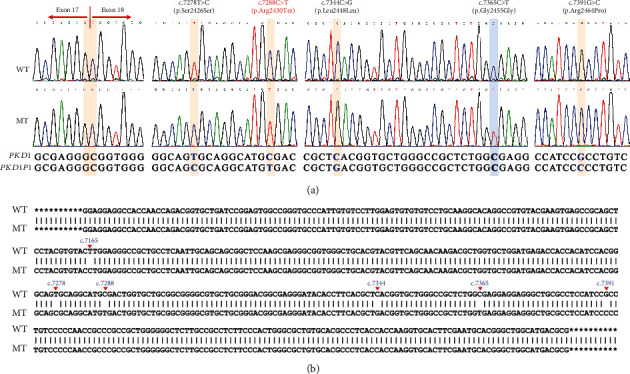
TA clone sequencing of RNA sample from proband. (a) The representative electropherograms of variants on Exon 18 of *PKD1*. The position of target variants were highlighted. Reference sequences of *PKD1* and *PKD1P1* were displayed at the bottom of the electropherograms, with differing bases marked in bold blue. The red arrow indicates the start of the junction between Exons 17 and 18 of *PKD1* gene. (b) Sequence alignment diagram of mutant clone and wild-type clone. Different bases were marked in bold blue and annotated with their positions on the *PKD1* gene. The asterisk indicates other bases that can be aligned but were not displayed. WT: wild type; MT: mutant type.

**Figure 4:**
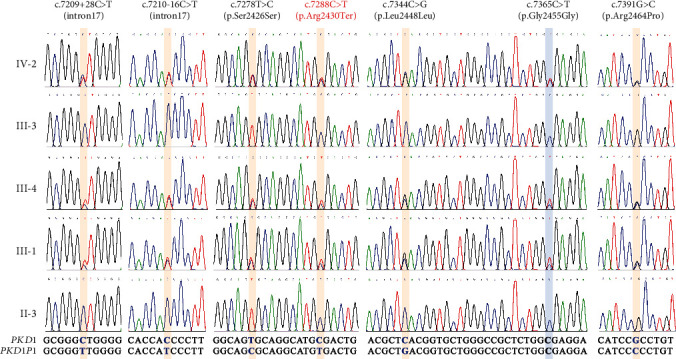
Pedigree analysis of variants by Sanger sequencing. The position of target variants were highlighted. The sequence of *PKD1* and *PKD1P1* was displayed at the bottom of the electropherograms, and the different base was marked as bold blue.

TA clone sequencing of the proband's RNA sample confirmed the presence of five heterozygous paralogous sequence variants (PSVs) on Exon 18 of *PKD1*, and all these PSVs were located at the same allele (Figure 3). Additionally, the sequences outside the mutated clonal conversion region can perfectly align with the wild-type *PKD1* (Figure 3(b)). The Sanger sequencing results of DNA samples from family members revealed that the seven PSVs detected by LRS were heterozygous in affected individuals (III-1, III-4, and IV-2). In contrast, unaffected individuals were homozygous for the wild type (II-3 and III-3) (Figure 4). Consequently, we confirmed that the gene conversion was inherited from the proband's mother, who had been diagnosed with PKD and received renal dialysis treatment at the age of 45.

## 4. Discussion

In this study, we conducted a genetic analysis on a family with three ADPKD patients. The proband was initially identified to carry a heterozygous deletion of Exons 17 and 18 and a heterozygous SNV in Exon 18 of *PKD1* by WES and MLPA (Figure 1). However, attempts to pinpoint the exact deletion breakpoint using Sanger sequencing were unsuccessful, as a smaller fragment was not obtained on agarose gel electrophoresis (data not shown). Subsequent sequencing of the long fragment recovered from the agarose gel revealed the presence of five heterozygous variants in Exon 18 of *PKD1* (Figure 1(c)). The discrepancy between the Sanger sequencing and the results of WES and MLPA prompted further investigation using LRS. The LRS analysis uncovered a gene conversion event involving 282 bp of *PKD1* originating from its pseudogenes, which encompassed the entire Exon 18 of *PKD1*.

A precise molecular diagnosis is crucial for accurate genetic counselling, prenatal diagnosis, and genotype–phenotype correlation analysis in patients with ADPKD [2]. However, genetic testing for ADPKD is challenging due to the presence of six pseudogenes that share 98% sequence similarity with *PKD1*, complicating Sanger sequencing and SRS approaches [7, 9]. WES is a timesaving and cost-effective method for screening numerous variants simultaneously, but its sensitivity can vary depending on the diseases, genes, and specific regions of the gene being analyzed [20]. Vaisitti et al. [6] have reported a positive rate of 56.5% (78/138) for determining the causative variant of monogenic kidney diseases using WES. Regarding *PKD1*, the sensitivity of WES for detecting mutations throughout the whole *PKD1* gene was 50%, but it dropped to 7.14% for mutations in *PKD1* Exons 1–32 [9]. Due to the high degree of homology between the pseudogenes and the genuine *PKD1*, as well as high GC content, the WES data showed reduced capture efficiency, low reading depth, and genotype quality and quality in Exons 1–32 of *PKD1* [9, 21, 22]. Furthermore, the probe capture methods in the presence of pseudogenes can result in complex variant calls and challenge the data interpretation within the target region [21], potentially leading to false positive results in some exceptional cases [23]. Therefore, the issues mentioned above might have contributed to the misidentification of Exons 17 and 18 deletions by WES in the present study.

As for the MLPA method, the presence of *PKD1* variants within the MLPA probe binding region of Exon 18 resulted in a false positive for Exon 18 deletion. It is known that the SNVs located in close proximity to the ligation sites can interfere with the hybridization or ligation of the MLPA probe, impacting the accuracy of the assay [24–26], and their impact varied from the proximity to the ligation sites [25, 26]. It was reported that SNVs <8 bp from the ligation sites can affect the hybridization or ligation of the MLPA probe, while the effects of SNVs beyond this distance were uncertain [25]. Specifically, the variant c.7344C>G was located at the 3′ end of the left probe oligonucleotide (LPO) (Table 2), resulting in a mismatch at the 3′end of LPO that hindered probe amplification [26, 27]. The variant c.7365C>T was located at 8 nt from the 3′ end of the right probe oligonucleotide (RPO) (Table 2), making its effect unpredictable. Nonetheless, the presence of the c.7344C>G variant was sufficient to cause the false positive result in MLPA analysis. Thus, this case emphasized the importance of validating the MLPA method, especially for examining single-exon deletion or duplication.

Recently, the rapidly developing LRS has emerged as a powerful tool for detecting repetitive or complex genomic regions, offering a significant advantage over the SRS method [10, 28]. This capability makes LRS well-suited for addressing the complex nature of *PKD1* and distinguishing the homologous regions from those of its pseudogenes. Borras et al. [11] reported that LRS-based LR-PCR amplification exhibited high sensitivity in identifying pathogenic variants in *PKD1*, successfully diagnosing 94.7% of the patients. They were able to identify 17 highly credible variants that had been missed by Sanger sequencing. Similarly, our result revealed that LRS-based hybridization capture identified a *PKD1* conversion event that has been missed by WES and MLPA techniques. Therefore, it seems that LRS could be used as an alternative strategy for the diagnosis of patients with ADPKD patients. However, further validation and evaluation are needed to assess the reliability and efficiency of LRS-based strategies as diagnostic tools for ADPKD.

Gene conversion is a mechanism known to generate pathogenic variants by replacing an allele in the genome with its homologous sequence, which has already been documented in various human Mendelian diseases [29, 30]. Although this phenomenon has been previously described in ADPKD caused by *PKD1* [29, 31], their exact genomic origin and extent have not been characterized. Rossetti et al. [32] recently reported a gene conversion event between *PKD1* and the duplicon *PKD1P6*, spanning over 8.5 kb and involving Exons 28–32. However, conventional SRS used by Rossetti et al. were unable to determine whether the PSVs were in *trans* or *cis*. Nonetheless, our LRS result directly identified the phase information of the PSVs being on the same parental alleles. This capability was particularly advantageous when parental segregation studies were not available. The unique characteristic of LRS, with its long read, enables the precise mapping of continuous haplotypes linking gene-specific regions to their pseudogene counterparts, facilitating the accurate delineation of the extent of gene conversion [8, 28]. In this study, the LRS results revealed that the maternal *PKD1* allele did not harbor a deletion of Exons 17 and 18 as previously suggested but instead underwent pseudogene-mediated gene conversion. This conversion event introduced several PSVs from *PKD1P1* to *PKD1*, including a known pathogenic variant (c.7288C>T), resulting in loss-of-function by introducing a premature stop codon in Exon 18 (p.Arg2430Ter).

In this study, we encountered certain limitations that impacted the definitive determination of the specific pseudogene involved in the gene conversion event with *PKD1*. The nucleotide sequences of *PKD1P1*, *PKD1P2*, and *PKD1P3* were found to be identical in the 282 bp minimal conversion region, making it challenging to ascertain which pseudogene underwent conversion with *PKD1*. Given this ambiguity, we chose to use *PKD1P1* as a representative in the manuscript for the sake of simplicity. Furthermore, we faced constraints in obtaining RNA samples from all family members due to undisclosed reasons. Despite this limitation, we were able to successfully verify the variants on Exon 18 of the *PKD1* gene in the proband's RNA sample.

In conclusion, our study utilized LRS to uncover a PKD pedigree caused by gene conversion in the *PKD1*, a finding that was initially misidentified by WES and MLPA. Our results identified the genetic etiology of the family and provided a basis for prenatal diagnosis and preimplantation genetic diagnosis. This case further highlighted the importance of validating CNVs or structural variants identified by WES through alternative methodologies, especially in complex structural regions like *PKD1*, where multiple approaches may be necessary. Moreover, we suggest that LRS might be more suitable than WES combined with MLPA for genetic screening of PKD, emphasizing the potential of LRS as a valuable tool in clinical diagnostics and research.

## Figures and Tables

**Table 1:** The list of primer sequences for Sanger sequencing.

**Name**	**Forward primer (5**′**→3**′**)**	**Reverse primer (5**′**→3**′**)**	**Fragment size (bp)**
*Genomic DNA*			
LR-PCR-DNA	GGCGATCACAGCGCAACTACT	ACGGAGTTGGCGGAGTTGGC	5316
Sequencing	TGGCAAACCGGATGAGTATC	TAGCTGGAGAGGCTGCC	918
*cDNA*			
LR-PCR-cDNA	AGCGCAACTACTTGGAGGCCC	ACCACAACGGAGTTGGCGG	2203

**Table 2:** Variants of exon with sequences of MLPA probes.

**Exon**	**Variants**	**LPO**	**RPO**
18	c.7344C>G	CGGGACGGCGAGGGATACACCTTCACGCTC	ACGGTGCTGGGCCGCTCTGGCGAGGAGG
18	c.7365C>T	CGGGACGGCGAGGGATACACCTTCACGCTC	ACGGTGCTGGGCCGCTCTGGCGAGGAGG

*Note:* Variants were underlined.

Abbreviations: LPO, left probe oligonucleotide; RPO, right probe oligonucleotide.

## Data Availability

The results of data generated and analyzed during this study are included in the article. Raw data from sequencing are not publicly available because they contain information that could compromise the privacy of study participants. Further enquiries can be directed to the corresponding author.
